# First Report of Chagas Disease Vector Species *Triatoma sanguisuga* (Hemiptera: Reduviidae) Infected with *Trypanosoma cruzi* in Delaware

**DOI:** 10.4269/ajtmh.23-0915

**Published:** 2024-03-26

**Authors:** Jennifer K. Peterson, Juliana Hoyos, Charles R. Bartlett, Nicole L. Gottdenker, Brian Kunkel, Carrie Murphy, Antonio Alvarado

**Affiliations:** ^1^ Department of Entomology and Wildlife Ecology, University of Delaware, Newark, Delaware;; ^2^ Odum School of Ecology, University of Georgia, Athens, Georgia;; ^3^ Department of Veterinary Pathology, University of Georgia College of Veterinary Medicine, Athens, Georgia;; ^4^ University of Delaware Cooperative Extension Service, Newark, Delaware;; ^5^ Delaware Department of Health and Social Services, Division of Public Health, Dover, Delaware

## Abstract

In July and October 2023, two live triatomine bugs were found inside a home in New Castle County, Delaware. The bugs were identified as *Triatoma sanguisuga*, the most widespread triatomine bug species in the United States. *Triatoma sanguisuga* is a competent vector of *Trypanosoma cruzi*, the causative agent of Chagas disease. The two specimens were tested via real-time PCR (qPCR) for infection with *T. cruzi*, and one of the specimens was positive. Despite *T. sanguisuga* being endemic to the area, attainment of accurate species identification and *T. cruzi* testing of the bugs required multiple calls to federal, state, private, and academic institutions over several months. This constitutes the first report of *T. sanguisuga* infected with *T. cruzi* in Delaware. In addition, this is the first published report of *T. sanguisuga* in New Castle County, the northernmost and most densely populated county in Delaware. New Castle County still conforms to the described geographic range of *T. sanguisuga*, which spans from Texas to the East Coast of the United States. The *T. cruzi* infection prevalence of the species has not been studied in the northeastern United States, but collections in southern states have found prevalences as high as 60%. The Delaware homeowner’s lengthy pursuit of accurate information about the vector highlights the need for more research on this important disease vector in Delaware.

## INTRODUCTION

Chagas disease is a vector-borne parasitic infection transmitted in the Americas by triatomine bugs (Hemiptera: Reduviidae: Triatominae). Caused by the protozoan *Trypanosoma cruzi*, Chagas disease is characterized by a short acute phase with flulike symptoms, followed by a lifelong, chronic phase that can lead to cardiac and gastrointestinal complications if left untreated.^1^ At least 6 million people are estimated to have Chagas disease globally, and annual mortality is approximately 10,000 people.^2^

Vector-borne *T. cruzi* transmission is stercoral (i.e., transmitted in bug excrement and not saliva). As a result, the likelihood of *T. cruzi* transmission from a single triatomine bite is low.^3^ Autochthonous Chagas disease cases most often occur in areas with triatomine bug species that are either domiciliated or highly intrusive.^4^ Of the 11 triatomine bug species in the United States, the most widespread is *Triatoma sanguisuga*. The species is found in at least 23 states from Texas to the eastern seaboard.^5^ Studies of *T. sanguisuga* in Florida,^6^ Texas,^7^^–^^9^ and Louisiana^10^^–^^12^ found that the species feeds on humans and multiple animal taxa^9^^,^^13^^,^^14^ in both rural and urban areas.^6^^,^^15^^,^^16^ *Trypanosoma cruzi* infection prevalences in *T. sanguisuga* collected in Louisiana and Texas between 2007 and 2016 ranged from 30% to 62%.^8^^,^^10^^,^^13^^,^^17^

*Triatoma sanguisuga* is found in all six states of the Mid-Atlantic (New Jersey, Pennsylvania, Delaware, Maryland, Virginia, and West Virginia) and Washington DC, but little is known of its regional ecology or epidemiology. Delaware was added to the species’ geographic range by the CDC in 2019, but entomological research collections in Delaware contain a small number of *T. sanguisuga* collected between 2004 and 2008, and citizen science reports exist from 2010 to the present day.^18^^–^^20^ *Triatoma sanguisuga* drew brief media attention in Kent County, Delaware, in 2018 when an adult specimen bit a child in her bedroom,^21^ but the species remains unstudied in the region.

## CASE DESCRIPTION

**Figure 1.**
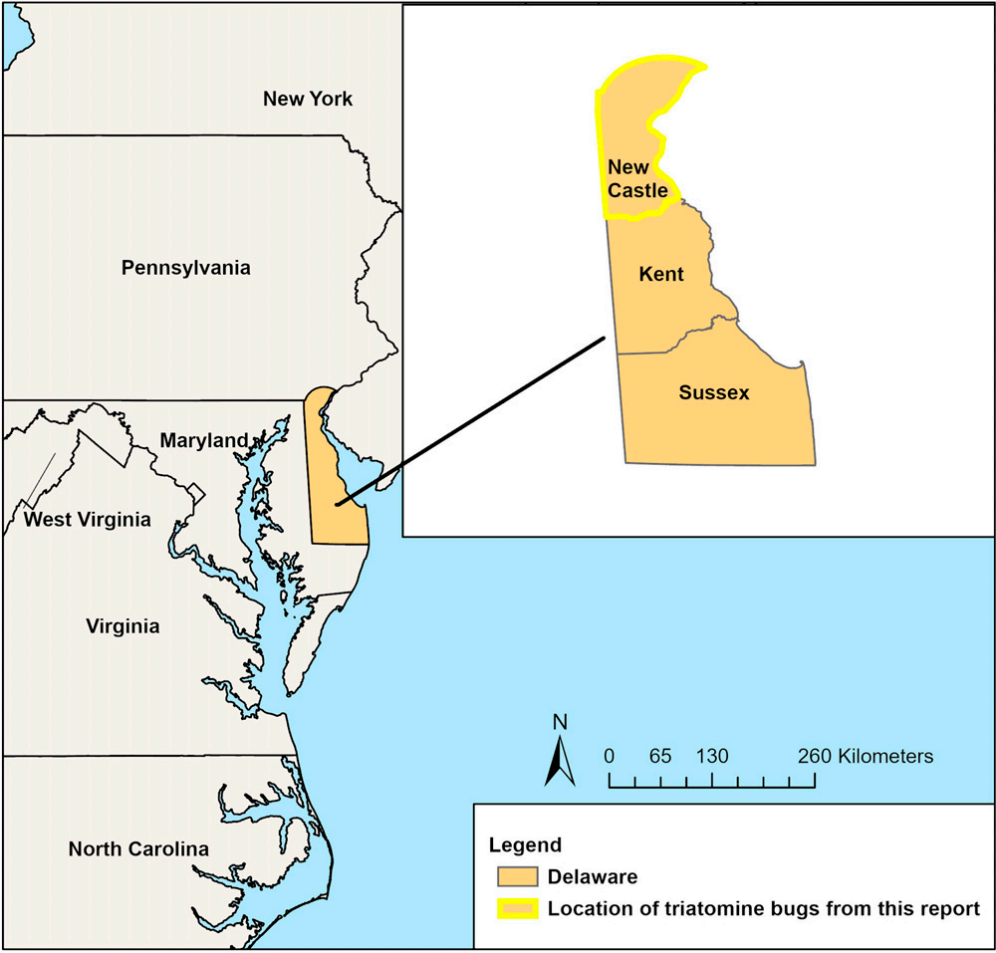
Three counties of Delaware with New Castle County highlighted in the inset.

**Figure 2.**
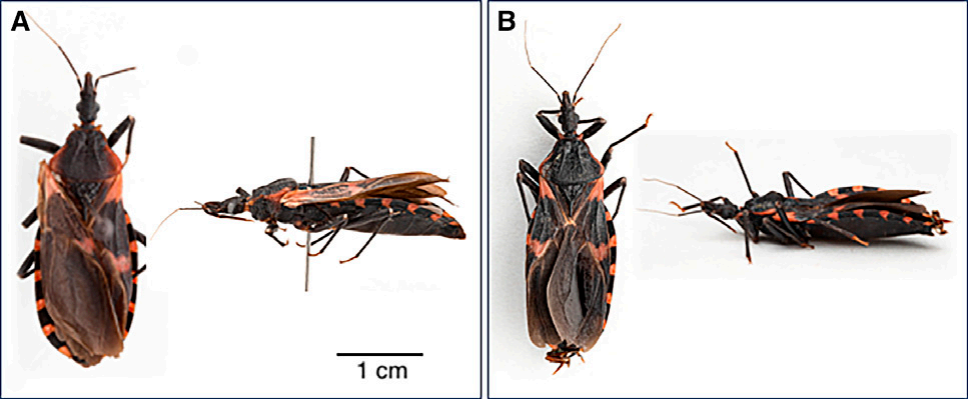
Dorsal and lateral views of each *Triatoma sanguisuga* specimen. (**A**) Specimen found in the master bedroom on July 7, 2023. (**B**) Specimen found in the kitchen on October 22, 2023. Photo taken by Solomon Hendrix.

In July and October of 2023, two live, adult, male *T. sanguisuga* were discovered in a New Castle County, Delaware, home (Figure 1). The first specimen (Figure 2A) was discovered at approximately 9:00 pm on July 7, 2023 in the master bedroom as the homeowner prepared to go to bed. The bug was sitting on their pillow under the comforter. The second specimen (Figure 2B) was found on a cookie sheet in the kitchen on the evening of October 22, 2023.

The house was a large single-family home with the master bedroom on the second floor and the kitchen on the first floor. A portion of the home was built in the 1700 s, but it was updated and well maintained. The foundation was a combination of brick and stone reinforced with concrete. Artificial lights (which are known to attract *T. sanguisuga*) were present on the exterior of the house, but all windows were covered with screens. The homeowner reported that due to the age of the home, there were likely crevices through which small insects could enter. A boric acid–based pesticide (Terro^®^ T2600, Lancaster, PA) was infrequently used around the home for ant control.

Residents consisted of four adults, one child, and two dogs. The dogs were described as indoor pets taken outside for walks only. The home was situated on a large estate with a pond and a horse pasture. The homeowner said they frequently see deer and foxes on the property and occasionally see squirrels and rabbits. The home was located less than 2 miles from highways in an area consisting of estates intermixed with farmland and forest fragments.

**Figure 3.**
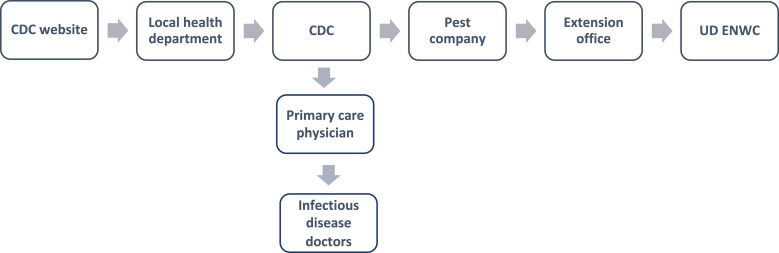
The process to obtain a species identification of a kissing bug found inside a home in Delaware. Each box is the entity that the prior entity recommended contacting. UDENWC = University of Delaware Department of Entomology and Wildlife Ecology.

The homeowner had found a large, unusual bite on their thigh 2 nights before finding the *T. sanguisuga* specimen in their bed. Another resident of the home had also noticed a similar bite on their body. The homeowner sought to confirm the identification of the bug and, if it was confirmed to be a kissing bug, to obtain testing for *T. cruzi*. In pursuit of this objective, the homeowner consulted the CDC website and then called the local health department, the CDC, a pest control company, a primary care physician, two infectious disease doctors, and a county extension office (Figure 3).

The homeowner also reached out to their primary care physician because they were experiencing heart palpitations. The physician called two infectious disease colleagues but received no response, so they administered a chest x-ray, electrocardiogram, and D-dimer to the homeowner. In the meantime, the bug was identified as a Hemipteran but not a triatomine, and the homeowner did not pursue further testing.

Two months later, the specimen came to the attention of faculty at the University of Delaware Department of Entomology and Wildlife Ecology, and it was identified by J.K. Peterson as *T. sanguisuga*.^22^ The homeowner was contacted immediately with the information, upon which they reported finding another specimen in their kitchen the previous night. We retrieved the second specimen, identified it as *T. sanguisuga*, and tested both specimens for *T. cruzi*. The homeowner again contacted their primary care physician to request *T. cruzi* testing. The physician connected the homeowner to an infectious disease doctor, who provided an order for *T. cruzi* antibody testing.

The *T. sanguisuga* specimens were tested for *T. cruzi* via real-time PCR (qPCR) as described in Muñoz-Calderón^23^ (Supplemental Figure 1). The *T. sanguisuga* specimen found in the homeowner’s bed (Figure 2A) was positive for *T. cruzi*. The specimen found in the kitchen (Figure 2B) was negative. Upon learning the bug in their bed tested positive for *T. cruzi,* the homeowner underwent *T. cruzi* antibody testing at a local commercial diagnostic laboratory (Labcorp). Available Labcorp documents indicate that they use the Alinity s Chagas test,^24^ which is a chemiluminescent microparticle immunoassay manufactured by Abbott™. The test result was negative.

## DISCUSSION

This is the first report of *T. sanguisuga* infected with *T. cruzi* in Delaware and the first published report of *T. sanguisuga* inside a home in New Castle County (NCC; Figure 1). Although NCC is the most densely populated and urban county in Delaware, the land use matrix of abutting farms, forest fragments, and human homes provides many blood meal sources for *T. sanguisuga,* which feeds opportunistically across multiple taxa. In a study of 45 bugs from coastal Louisiana (where hundreds of specimens of *T. sanguisuga* have been collected^10^^,^^11^^,^^13^^,^^25^), predominant blood meal sources identified were tree frogs (53%), humans (48%), and raccoons (30%), all of which are abundant in Delaware^13^ (much of which is also coastal). Interestingly, the homeowner in this report had never seen raccoons or opossums on their property. However, other triatomine species are known to fly upwards of a kilometer^26^^–^^28^; assuming the same holds true for *T. sanguisuga* in Delaware, the bugs likely reproduce in animal nests and burrows in forest patches and then fly to nearby human homes to forage. We expect this trend to continue as housing developments and other land conversion continue to encroach on natural habitats.

Historically, domestic triatomines were associated with rural houses constructed with natural materials, as well as with certain animal husbandry practices and piles of wood and junk.^29^ Today several triatomine populations are adapted to urban and peri-urban environments.^15^^,^^30^^–^^34^ Of the U.S. species, *T. rubida* and *T. sanguisuga* seem adept at entering human homes regardless of the age or integrity of the home.^17^^,^^35^ Indeed, the house and yard in this report did not conform to conventional triatomine bug risk factors; they were well constructed, well maintained, and had screens on the windows. Other possible risk factors could be the presence of artificial lights and livestock. A better understanding of the regionally specific risk factors for *T. sanguisuga* in Delaware will help to resolve this question and identify preventative measures for future *T. sanguisuga* home intrusions. Integrated pest management strategies that target sociocultural, biological, and physical risk factors using a combination of education, structural modifications, and pesticides have proven effective against *T. sanguisuga* in the southern United States.^36^

The presence of a *T. cruzi*–infected *T. sanguisuga* in the homeowner’s bed is concerning, albeit unsurprising, given the *T. cruzi* infection rates in the species elsewhere in its range. In addition, Delaware is home to many mammal species that maintain the *T. cruzi* transmission cycle, namely the North American opossum (*Didelphis virginiana*) and the common raccoon (*Procyon lotor*).^37^^–^^41^ A 1958 study revealed *T. cruzi* infections in five raccoons captured in Maryland,^42^ but to the best of our knowledge, the subject has not been studied since. More research is needed to identify animal hosts for *T. cruzi* in Delaware, including synanthropic species that could serve as sentinels hosts for the presence of both the bug and the parasite.

Finally, the homeowner’s experience in this report demonstrates the importance of widely available public health resources on endemic vector species in the United States, including how to identify a vector and recommended actions when encountering a vector in your home. Locally acquired vector-borne *T. cruzi* infection in humans is rarely reported in the United States, likely because it occurs infrequently, but also because few people are looking (just six states have Chagas surveillance or reporting systems^43^) and awareness among medical professionals is low.^44^ *Triatoma sanguisuga* was implicated as the vector species involved in autochthonous Chagas disease cases in Louisiana,^45^ Tennessee,^46^ and Mississippi,^47^ yet virtually nothing is known about the species its northern range. More research into domestically invasive triatomines in northern states could help raise public awareness and in turn facilitate testing of individuals with known triatomine exposures, as recommended by Forsyth et al.^48^ Such screenings could increase the potential to diagnose autochthonous Chagas cases early enough to successfully treat the disease.

## CONCLUSION

This case illustrates a public health resource gap regarding *T. sanguisuga* in Delaware. Although Chagas disease is not of highest concern in the United States, other infrequently transmitted vector-borne diseases, such as mosquito-transmitted encephalitis viruses, are actively studied, surveilled, and reportable in the northeast. Similar treatment of *T. sanguisuga* in the Mid-Atlantic region could be used to determine risk factors for vector encounters, inform public education efforts, and develop resources for citizens to consult should they encounter a vector in their home or elsewhere.

## Supplemental Materials

10.4269/ajtmh.23-0915 Supplemental Materials
