# Supplementary material for: First Report of Chagas Disease Vector Species Triatoma sanguisuga (Hemiptera: Reduviidae) Infected with Trypanosoma cruzi in Delaware
Source: Am J Trop Med Hyg. 2024 Mar 26;110(5):925–9. doi: 10.4269/ajtmh.23-0915 (PMC11066352; doi:10.4269/ajtmh.23-0915)
Supplement: Supplemental Materials [file tpmd230915.SD1.pdf]

Supplementary materials

S1. DNA extraction and RT-PCR

Genomic DNA was extracted from the intestinal contents of each bug using a phenol-chloroform procedure[1]. The genomic DNA was then run in a duplex TaqMan qPCR that amplifies a 175bp sequence of the 24S alpha subunit of the T. cruzi rDNA gene[2]. Quantitative real-time PCR was performed on a CFX384 Touch Real-time PCR cycler (Bio-Rad) using the FastStart Universal Probe Master (Roche) according to the manufacturer's instructions. The reaction mix for all reactions consisted of 10 µl FastStart Universal Probe Master, 0.25 µl HEX-labelled probe, 0.15 µL FAM-labelled probe, 1.8 µl D75b primer, 1.8 µl D76b primer, 3 µl water, and 3 µl genomic DNA. Samples were run in duplicate with negative and positive controls. Cycling conditions were an initial step of 10 min at 95 °C and 40 cycles at 95 °C for 30s and 60 °C for 1 min.

T. cruzi was detected for both runs of the DNA from that specimen, with quantification cycle (C_q) values of 31.1 and 31.3; whereas C_q values for the positive controls were 25 and 29.58 (Fig. S1). These values indicate the number of cycles run at which the target sequence is detected in the assay, and generally speaking, C_q values under 37 are considered positive.

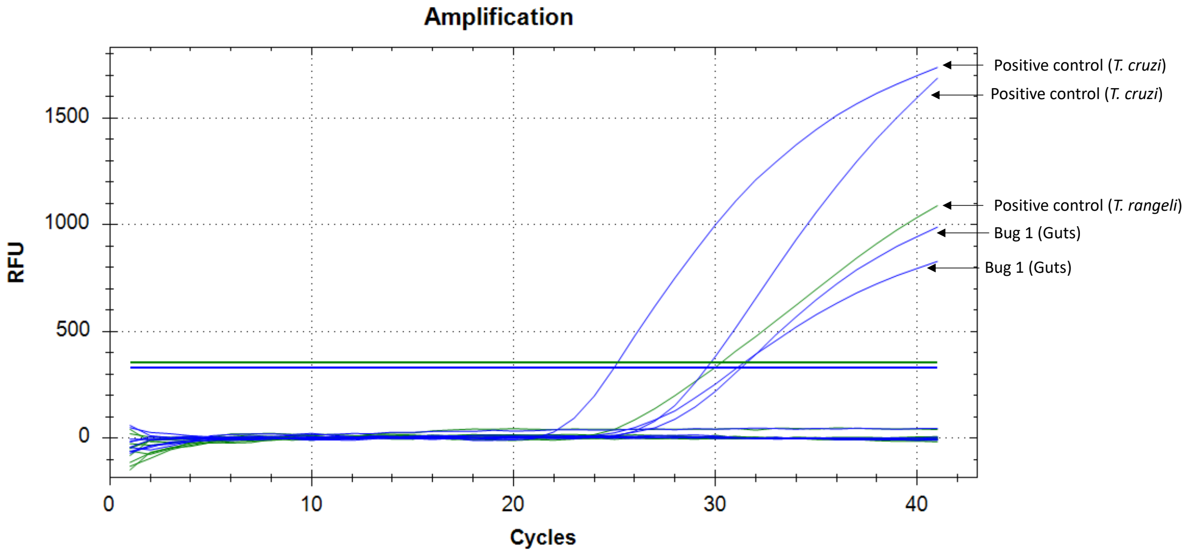

Figure S1. RT-PCR amplification curves for DNA extracted from the *T. sanguisuga* found in the homeowner's bed ('Bug 1").

References

1. Kieran TJ, Gottdenker NL, Varian CP, Saldaña A, Means N, Owens D, Calzada JE, Glenn TC., 2017. Blood Meal Source Characterization Using Illumina Sequencing in the Chagas Disease Vector Rhodnius pallescens (Hemiptera: Reduviidae) in Panamá. *Journal of Medical Entomology 54*: 1786–1789

2. Muñoz-Calderón A, Wehrendt D, Cura C, Gómez-Bravo A, Abril M, Giammaria M, Lucero RH, Schijman AG., 2020. Real-time polymerase chain reaction based algorithm for differential diagnosis of Kinetoplastidean species of zoonotic relevance. *Infection, Genetics and Evolution 83*: 104328
